# International multi-center study to quantify the effect of deep venous drainage after surgical resection of Spetzler-Martin Grade II-III brain arteriovenous malformations

**DOI:** 10.1007/s10143-026-04310-0

**Published:** 2026-05-22

**Authors:** Avi A. Gajjar, Rashad Jabarkheel, Mohamed M. Salem, Basel Musmar, Sandeep Kandregula, Hammam Abdalrazeq, Nimer Adeeb, Assala Aslan, Nathan Ramachandran, Stavropoula I Tjoumakaris, Hamza Adel Salim, Adam A. Dmytriw, Christopher S. Ogilvy, Mustafa K. Baskaya, Douglas Kondziolka, Jason Sheehan, Howard Riina, Abdallah Abushehab, Kareem El Naamani, Najib Muhammad, Ahmed Abdelsalam, Natasha Ironside, Deepak Kumbhare, Sanjeev Gummadi, Cagdas Ataoglu, Muhammed Amir Essibayi, Abdullah Keles, Sandeep Muram, Daniel Sconzo, Arwin Rezai, Omar Alwakaa, Pierce Davis, Salem M Tos, Ufuk Erginoglu, Johannes Pöppe, Rajeev D. Sen, Alan S. Boulos, John C. Dalfino, Christoph J. Griessenauer, Robert M. Starke, Laligam N. Sekhar, Michael R. Levitt, David J. Altschul, Neil Haranhalli, Malia McAvoy, Hussein A. Zeineddine, Adib A. Abla, Saman Sizdahkhani, Sravanthi Koduri, M. Reid Gooch, Robert H. Rosenwasser, Christopher Stapleton, Matthew Koch, Peng R. Chen, Spiros Blackburn, Ketan Bulsara, Louis J. Kim, Omar Choudhri, Bryan Pukenas, Joshua S. Catapano, Darren Orbach, Edward Smith, Pascal J. Mosimann, Alexandra R. Paul, Pascal Jabbour, Ali Alaraj, Mohammad A. Aziz-Sultan, Aman B. Patel, Amey Savardekar, Christina Notarianni, Hugo H. Cuellar, Bharat Guthikonda, Jacques Morcos, Michael Lawton, Jan-Karl Burkhardt, Visish M. Srinivasan

**Affiliations:** 1 Department of Neurosurgery, Hospital of the University of Pennsylvania (HUP), Penn Medicine, 3400 Civic Center Blvd, Philadelphia, PA 19104 USA; 2 Department of Neurosurgery, Albany Medical College, Albany, NY USA; 3 Department of Neurosurgery, Thomas Jefferson University Hospital, Philadelphia, PA USA; 4 Department of Radiology, Louisiana State University, Shreveport, LA USA; 5 Department of Neurosurgery, Louisiana State University Health Science Center, Shreveport, LA USA; 6 Department of Neurosurgery, UT Health Sciences Center at Houston, McGovern Medical School, Houston, TX USA; 7 Neuroendovascular Program, Massachusetts General Hospital, Harvard Medical School, Boston, MA USA; 8 Neurosurgical Service, Beth Israel Deaconess Medical Center, Harvard Medical School, Boston, MA USA; 9 Department of Neurosurgery, University of Wisconsin School of Medicine, Madison, WI USA; 10 Department of Neurosurgery, New York University Grossman School of Medicine, New York, NY USA; 11 Department of Neurosurgery, University of Virginia, Charlottesville, VA USA; 12 Department of Plastic Surgery, Mayo Clinic Hospital, Rochester, MN USA; 13 Department of Neurosurgery, University of Miami, Miller School of Medicine, Miami, FL USA; 14 Montefiore Einstein Cerebrovascular Research Lab, Department of Neurological Surgery, Montefiore Medical Center, Albert Einstein College of Medicine, Bronx, NY USA; 15 Department of Neurosurgery, Christian Doppler Klinik, Paracelsus Medical University, Salzburg, Austria; 16 Department of Neurosurgery, University of Washington, Seattle, WA USA; 17 Department of Neurosurgery, University of Florida, Gainesville, FL USA; 18 Department of Neurosurgery, University of Connecticut, Farmington, CT USA; 19 Neurointerventional Radiology, Boston Children’s Hospital, Harvard Medical School, Boston, MA USA; 20 Department of Neurosurgery, Boston Children’s Hospital, Harvard Medical School, Boston, MA USA; 21 Division of Interventional and Diagnostic Neuroradiology, Department of Radiology, University of Toronto & Toronto Western Hospital, Toronto, Canada; 22 Department of Neurosurgery, University of Illinois in Chicago, Chicago, IL USA; 23 Department of Neurosurgery, Brigham and Women’s Hospital, Harvard Medical School, Boston, MA USA; 24 Department of Neurosurgery, Barrow Neurological Institute, Phoenix, AZ USA

**Keywords:** Arteriovenous malformations, DVD, Spetzler-Martin grading, Surgical outcomes, mRS, AVMs

## Abstract

Deep venous drainage (DVD) is considered a negative prognostic factor in AVM surgery, yet its effect on postoperative functional decline remains incompletely defined. This study evaluates whether DVD predicts worsened functional status after surgical resection of Spetzler-Martin Grade II-III AVMs. This retrospective multicenter study analyzed 129 patients with Spetzler-Martin Grade II-III AVMs across nine centers in North America and Europe who underwent primary surgical resection. We excluded cases with prior endovascular or stereotactic interventions. The primary outcome measured was poor functional status, defined as modified Rankin Scale (mRS) score 3–6 at last follow up. Among 129 patients with Spetzler-Martin Grade II-III AVMs, 38 (29.5%) exhibited deep venous drainage (DVD). Poor functional outcome (mRS ≥ 3) at last follow-up occurred in 14 patients (10.9%). This occurred in 6 of 38 patients with DVD (15.8%) compared with 8 of 91 without DVD (8.8%; Fisher’s exact *p* = 0.244). On univariate Firth-penalized logistic regression, DVD was not significantly associated with poor outcome (OR 1.96, 95% CI 0.65–5.89; *p* = 0.228). In the primary reduced Firth model adjusting for age and pre-existing functional disability, DVD was independently associated with poor outcome (OR 6.87, 95% CI 1.07–44.20; *p* = 0.042). Increasing age (OR 1.08 per year, 95% CI 1.02–1.13; *p* = 0.004) and pre-existing functional disability (OR 6.53, 95% CI 1.63–26.22; *p* = 0.008) were also independently associated with poor outcome. DVD is associated with functional decline following surgical resection of Spetzler-Martin Grade II-III AVMs after adjustment for age and pre-existing functional disability.

## Introduction

Arteriovenous malformations (AVMs) are complex vascular lesions characterized by abnormal, direct connections between arteries and veins, which bypass capillary networks and result in high-pressure, high-flow shunting, predisposing patients to intracranial hemorrhage [1]. Surgical resection is often cited as the gold standard for definitive AVM treatment, as it fully eliminates the hemorrhagic risk associated with these lesions [2–4]. Key factors, including AVM size, venous drainage patterns, and proximity to critical brain areas, guide surgical risk assessment and decision-making. These were defined by Robert Spetzler and Neil Martin in 1986, in a seminal paper where they proposed the Spetzler-Martin (SM) grading system, an internationally used metric of surgical risk assessment [5–10]. The SM grade is calculated based on AVM size, location (eloquent vs. noneloquent), and presence of deep venous drainage, with higher scores indicating greater surgical risk [11]. 

This system classifies AVMs into five grades, ranging from Grade I, representing small, superficial AVMs in non-critical brain regions, to Grade V, which includes large, deep-seated lesions in eloquent areas essential for neurological function. Grade V AVMs are frequently deemed inoperable [12]. 

While deep venous drainage (DVD) has been associated with an elevated risk of hemorrhagic presentation and AVM recurrence, its true impact on surgical outcomes remains unclear [13–15]. Existing literature demonstrates a correlation between increasing SM grade and heightened surgical risk [16]. Given conflicting reports, we aimed to quantify the impact of DVD on postoperative functional decline. The primary objective of this study is to evaluate the influence of DVD on surgical outcomes in primary surgical AVM resection.

## Methods

### Data source

This study utilized data from the Multicenter International Study for Treatment of Brain AVMs (MISTA) consortium, which includes cases from academic institutions across North America and Europe. Additional centers were invited to contribute their cases. A total of nine contributing centers that had Spetzler-Martin Grade II or III AVMs were included. Data were collected using a standardized form that captured comprehensive patient information, including demographics (e.g., age, gender, race, smoking history, comorbid conditions, and presence of hereditary disorders such as hereditary hemorrhagic telangiectasia or Sturge-Weber syndrome), clinical presentation, modified Rankin Scale (mRS) scores, and antiplatelet medication use. Detailed AVM characteristics (e.g., lesion side, size, location, Spetzler-Martin grade, nidus compactness, feeder and draining vein status, associated aneurysms, vascular anomalies, and any prior treatment) were documented along with procedural specifics (e.g., treatment date, type, and technical details). Data on complications, angiographic, and functional outcomes (e.g., follow-up imaging duration, obliteration rates, recurrences, and follow-up clinical mRS scores) were meticulously recorded. Senior neurosurgeons or neuro-interventionalists at each site verified AVM characteristics, complications, and outcome classifications to ensure consistency.

Institutional Review Board approval was obtained from all participating institutions. Patient data remained de-identified, and a designated coordinator at each site was responsible for ensuring data integrity and accuracy. All data were consolidated into a central, secure database, safeguarded on a password-protected device that was accessible only to authorized personnel. Patients were retrospectively identified from institutional surgical databases at each participating center over the study period, 2014–2025. While cases were drawn from surgical records, strict consecutive enrollment could not be verified across all sites. This study followed the Strengthening the Reporting of Observational studies in Epidemiology (STROBE) guidelines.

### Patient selection and statistical analysis

**Fig. 1:**
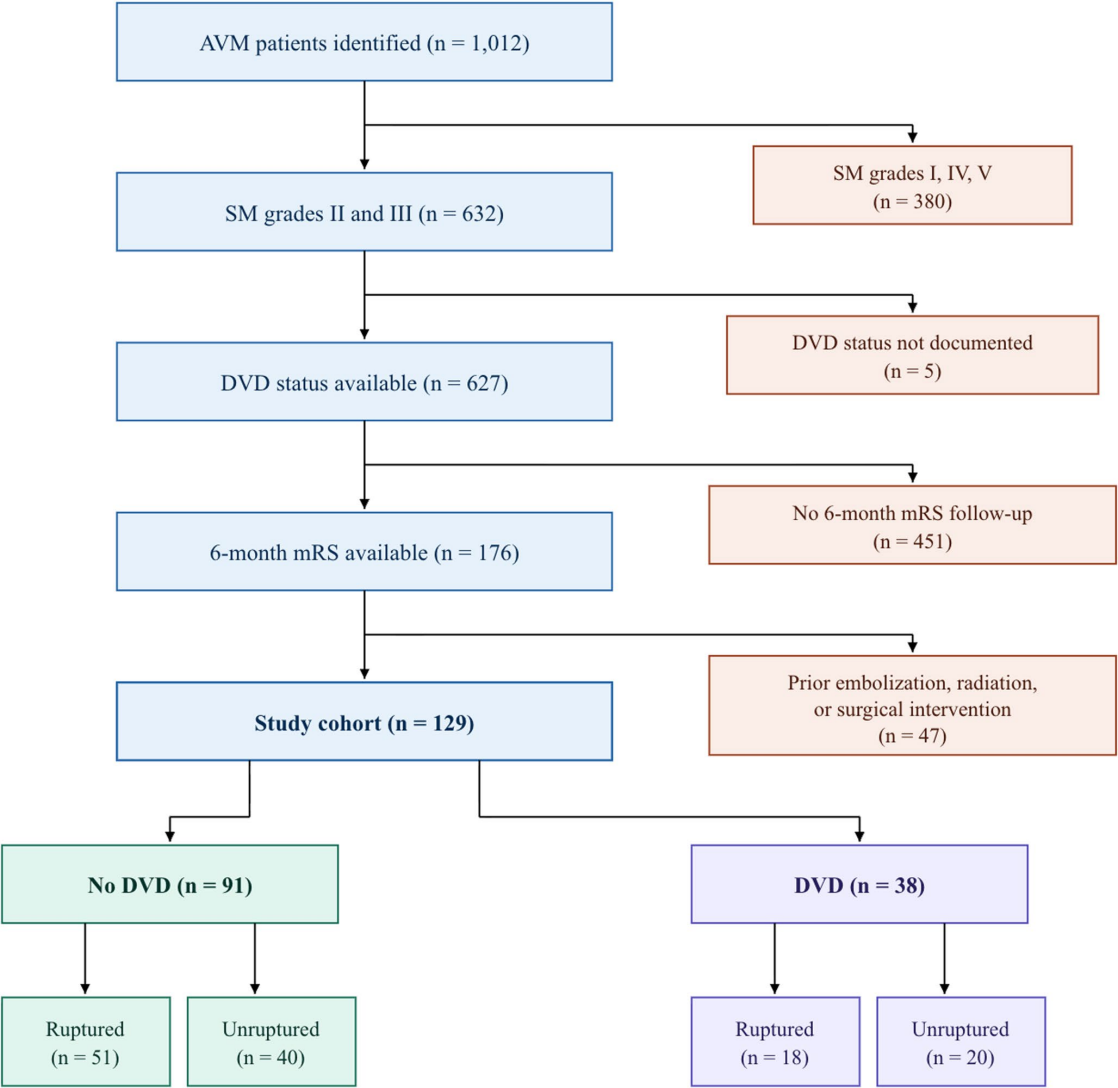
Flowchart diagram of AVM patient inclusion criteria

We included patients with Spetzler-Martin Grade II or III AVMs who underwent primary surgical treatment (Fig. 1). To maintain consistency, we excluded patients who had received prior embolization, radiation, or surgical intervention before the index procedure. The cohort comprised both ruptured and unruptured AVM cases. The primary outcome measure was poor mRS, defined as an mRS score of 3–6 at last follow-up. DVD was defined as the presence of any drainage into the deep venous system (including internal cerebral veins, basal veins of Rosenthal, vein of Galen, or subependymal veins), consistent with Spetzler-Martin grading criteria. Lesions with either purely deep or mixed superficial and deep drainage were classified as DVD.

Univariable associations were assessed using Firth-penalized logistic regression, which applies a bias-reducing penalty to the likelihood function and is recommended for rare-events data where standard maximum-likelihood estimates may be unreliable. Given the limited number of outcome events, a tiered modeling strategy was employed to maintain adequate event-per-variable (EPV) ratios. The primary (reduced) model included DVD, age, and pre-existing functional disability (EPV = 4.7). A sensitivity analysis added SM Grade III and rupture status (intermediate model, EPV = 2.8). More complex models were not pursued due to the limited number of EPV. Collinearity was assessed using variance inflation factors (VIF). Median follow-up duration was compared between groups using the Wilcoxon rank-sum test [17]. Pre-existing functional disability was classified as pre-operative mRS > 2. A p-value < 0.05 was considered statistically significant. All analyses were performed using Stata SE Version 18.0 (StataCorp, College Station, TX).

## Results

### Demographics

A total of 129 patients with Spetzler-Martin Grade II or III AVMs were included (Table 1). Of these, 38 patients (29.5%) demonstrated deep venous drainage (DVD), while 91 (70.5%) did not. The cohort was predominantly male (65 patients, 52.8%), with a mean age of 46.1 ± 19.8 years. Most AVMs were Grade II (93 patients, 72.1%). Compact morphology was observed in 81 patients (71.7%), and diffuse AVMs in 32 (28.3%). Ruptured presentation occurred in 69 patients (53.5%), and pre-existing functional disability was present in 42 patients (32.6%). Patients with DVD were significantly younger than those without DVD (39.1 ± 18.8 vs. 49.0 ± 19.6 years, *p* = 0.009). No significant differences were observed in sex, race, smoking status, compactness, or rupture status between groups (all *p* > 0.05).

**Table 1:** Demographic Variables of Patients by DVD

	No DVD	DVD	Total	Test
*N*	91 (70.5%)	38 (29.5%)	129 (100.0%)	
Age	49.033 (19.618)	39.132 (18.835)	46.116 (19.842)	**0.009**
Sex				
Male	47 (54.7%)	18 (48.6%)	65 (52.8%)	0.541
Female	39 (45.3%)	19 (51.4%)	58 (47.2%)	
Race				
Caucasian	62 (69.7%)	27 (71.1%)	89 (70.1%)	0.405
African American	15 (16.9%)	5 (13.2%)	20 (15.7%)	
Hispanic	7 (7.9%)	6 (15.8%)	13 (10.2%)	
Asian	3 (3.4%)	0 (0.0%)	3 (2.4%)	
Other	2 (2.2%)	0 (0.0%)	2 (1.6%)	
Smoking				
Current	27 (30.0%)	13 (34.2%)	40 (31.2%)	0.177
Former	16 (17.8%)	2 (5.3%)	18 (14.1%)	
Never	47 (52.2%)	23 (60.5%)	70 (54.7%)	
SM Grade				
2	79 (85.9%)	14 (37.8%)	93 (72.1%)	**< 0.001**
3	13 (14.1%)	23 (62.2%)	36 (27.9%)	
Compacted				
0	19 (24.4%)	13 (37.1%)	32 (28.3%)	0.163
1	59 (75.6%)	22 (62.9%)	81 (71.7%)	
Rupture				
0	40 (44.0%)	20 (52.6%)	60 (46.5%)	0.368
1	51 (56.0%)	18 (47.4%)	69 (53.5%)	

### Poor outcome at last follow-up

At last follow-up, 14 patients (10.9%) experienced poor functional outcome (mRS 3–6), while 115 (89.1%) had favorable outcomes (mRS 0–2) (Table 2). Patients with poor outcome were significantly older (62.1 ± 13.0 vs. 44.2 ± 19.7 years, *p* = 0.001) and more likely to have ruptured AVMs (78.6% vs. 50.4%, *p* = 0.046) and pre-existing functional disability (71.4% vs. 27.8%, *p* = 0.001). Compact morphology demonstrated a trend toward association with poor outcome (92.9% vs. 68.7%, *p* = 0.060). DVD was more frequent among patients with poor outcome (42.9% vs. 27.8%), although this difference was not statistically significant (*p* = 0.244). On univariate Firth-penalized logistic regression (Table 3), increasing age was significantly associated with poor functional outcome (OR 1.06, 95% CI 1.02–1.10, *p* = 0.004). No other variables, including DVD (OR 1.96, 95% CI 0.65–5.89, *p* = 0.228), rupture status, SM grade, compactness, sex, race, or smoking status, were significantly associated with poor outcome.

**Table 2:** Demographics by mRS on Last Follow Up

	mRS 0–2	mRS 3–6	Total	Test
*N*	115 (89.1%)	14 (10.9%)	129 (100.0%)	
Deep				
0	83 (72.2%)	8 (57.1%)	91 (70.5%)	0.244
1	32 (27.8%)	6 (42.9%)	38 (29.5%)	
Age	44.174 (19.688)	62.071 (13.029)	46.116 (19.842)	**0.001**
Sex				
Male	59 (54.1%)	6 (42.9%)	65 (52.8%)	0.426
Female	50 (45.9%)	8 (57.1%)	58 (47.2%)	
Race				
Caucasian	80 (70.2%)	9 (69.2%)	89 (70.1%)	0.885
African American	17 (14.9%)	3 (23.1%)	20 (15.7%)	
Hispanic	12 (10.5%)	1 (7.7%)	13 (10.2%)	
Asian	3 (2.6%)	0 (0.0%)	3 (2.4%)	
Other	2 (1.8%)	0 (0.0%)	2 (1.6%)	
Smoking				
Current	37 (32.2%)	3 (23.1%)	40 (31.2%)	0.185
Former	14 (12.2%)	4 (30.8%)	18 (14.1%)	
Never	64 (55.7%)	6 (46.2%)	70 (54.7%)	
SM Grade				
2	**84 (73.0%)**	**9 (64.3%)**	**93 (72.1%)**	0.521
3	**31 (27.0%)**	**5 (35.7%)**	**36 (27.9%)**	
Compactness	68 (68.7%)	13 (92.9%)	81 (71.7%)	0.060
Ruptured	58 (50.4%)	11 (78.6%)	69 (53.5%)	**0.046**
Pre-Existing Functional Disability	32 (27.8%)	10 (71.4%)	42 (32.6%)	**0.001**

**Table 3:** Univariable Firth-penalized logistic regression of mRS at last follow-up being 3–6

	OR	95% CI	*p*-val
DVD	1.96	0.65–5.89	0.228
Age	1.06	1.02–1.10	**0.004**
Sex			
Male		Reference	
Female	1.54	0.52–4.57	0.436
Race			
Caucasian		Reference	
African American	1.69	0.45–6.41	0.437
Hispanic	1.02	0.16–6.29	0.986
Smoking	1.21	0.06–25.25	0.902
Never	1.69	0.08–38.02	0.74
Current			
Former		Reference	
SM Grade III	3.33	0.73–15.25	0.122
Compact	1.08	0.28–4.21	0.912
Ruptured	1.42	0.46–4.40	0.539
Pre-existing Functional Disability	4.14	0.73–23.55	0.109

In the primary reduced Firth-penalized logistic regression model (Table 4), increasing age (OR 1.08, 95% CI 1.02–1.13, *p* = 0.004), deep venous drainage (OR 6.87, 95% CI 1.07–44.20, *p* = 0.042), and pre-existing functional disability (OR 6.53, 95% CI 1.63–26.22, *p* = 0.008) were independently associated with poor functional outcome. Rupture status (OR 3.85, *p* = 0.101), SM Grade III (OR 1.52, *p* = 0.612), and compact morphology (OR 3.23, *p* = 0.245) were not independently associated with outcome.

Variance inflation factors were low across all predictors (VIF ≤ 1.42), indicating no evidence of multicollinearity. Deep venous drainage was moderately correlated with SM Grade III (*r* = 0.456, *p* < 0.001), consistent with its role in Spetzler-Martin grading, but was not associated with rupture status (*p* = 0.602).

**Table 4:** Firth-penalized logistic regression

Variable	OR	95% CI	*p*-value
Age	**1.08**	**1.02–1.13**	**0.004**
Ruptured	3.85	0.77–19.27	0.101
SM Grade III	1.52	0.30–7.61	0.612
Compacted	3.23	0.45–23.41	0.245
Deep Venous Drainage	**6.87**	**1.07–44.20**	**0.042**
Pre-existing Functional Disability	**6.53**	**1.63–26.22**	**0.008**

## Discussion

DVD is independently associated with postoperative functional decline in patients with Spetzler-Martin Grade II-III arteriovenous malformations. After adjustment for age and pre-existing functional disability, DVD was associated with a nearly seven-fold increase in the odds of poor functional outcome at last follow-up (OR 6.87, 95% CI 1.07–44.20; *p* = 0.042). Notably, DVD was not significantly associated with outcome on univariate analysis (OR 1.96, *p* = 0.228). The emergence of significance after adjustment likely is consistent with negative confounding by age, as patients with DVD were significantly younger, while increasing age independently predicted poor outcome.

### Theory behind a theoretical protective effect of DVD

Several hemodynamic models suggest that DVD can moderate intranidal pressure by providing a low-resistance venous pathway. In in vitro simulations and canine fistula models, lesions with deep outflow exhibited lower transnidal pressure gradients and attenuated surges when systemic blood pressure rose, implying a buffer against sudden stress on the nidus [18]. Computational flow analyses reached similar conclusions that larger, centrally draining nidi redistributed venous pressures more evenly, potentially reducing vessel wall tension and the propensity for rupture [19]. Draining-vein pressure measurements further support this concept; although vein pressure rises in parallel with arterial inflow, the arterial-venous gradient narrows when deep channels are available, indicating compensatory modulation rather than straightforward transmission of arterial load [18]. 

The theoretical protective effect of DVD may reflect the presence of preserved deep venous egress during perioperative hemodynamic perturbations. Drainage through deep medullary, subependymal, basal, or internal cerebral venous pathways could provide additional outflow capacity when superficial cortical drainage is limited, compressed, or altered during surgery. Maintaining venous decompression of the nidus may hypothetically attenuate transient increases in venous pressure and reduce abrupt changes in nidus wall stress during anesthetic induction, hypercapnia, vasopressor exposure, or operative manipulation.

Clinical evidence, however, does not corroborate a net benefit. In our cohort, DVD was associated with approximately a sevenfold increase in adjusted odds of poor mRS, and this association was consistent across sensitivity analyses incorporating additional covariates. Because DVD is a component of the Spetzler-Martin grading system and was correlated with Grade III status (*r* = 0.456, *p* < 0.001), we performed stratified analyses by SM grade; although cell sizes precluded formal regression, the direction of effect was consistent within both Grade II and Grade III subgroups. The adverse association with DVD likely stems from technical challenges as deep veins lie in eloquent regions, resist bipolar coagulation, and, if sacrificed prematurely, precipitate venous hypertension, nidus congestion, and catastrophic rupture. Moreover, a larger nidus size and central brain location often coexist, each being an additional risk factor for a poor outcome.

### Comparison to established literature and the Spetzler-Martin grading system

These findings refine the Spetzler-Martin paradigm by quantifying the extent to which DVD worsens outcomes in Grade II-III AVMs, despite historical assumptions that its impact may be modest [12]. DVD has traditionally been perceived as a complicating factor, potentially increasing intraoperative difficulty and postoperative morbidity due to the fragility and retractability of deep veins, which can bleed into critical areas if disrupted. As Spetzler and Martin observed, these veins resist standard coagulation techniques and may create challenges during AVM excision, particularly in lesions adjacent to eloquent brain regions.

However, advancements in neurosurgical techniques, such as improved bipolar coagulation and enhanced microsurgical tools, have likely helped mitigate some of the traditional risks associated with DVD [20, 21]. Additionally, modern approaches to deep-seated brain lesions, such as those used for brainstem cavernous malformations, have increasingly employed safe entry zones, enabling access to previously inaccessible brain regions with minimized collateral damage [22, 23]. Surgical handling of AVMs with DVD requires meticulous preservation of venous outflow until the nidus is fully devascularized to avoid catastrophic venous infarction or nidus rupture. Techniques such as circumferential dissection of the nidus, progressive obliteration of arterial feeders, and delayed occlusion of deep veins are critical to managing these lesions safely. These advances partially explain our findings and suggest that the Spetzler-Martin grading system could benefit from modifications incorporating current surgical innovations, particularly regarding the resectability of Grade II and III AVMs with DVD.

Moreover, recent studies support that DVD does not uniformly elevate surgical risks. In subgroup analyses by Davidson and Morgan, Grade II AVMs with DVD did not show a significant increase in adverse surgical outcomes compared to similar AVMs without deep drainage [16]. Size and location-based studies also indicate that DVD is not associated with worse outcomes in specific configurations, suggesting that the anatomical nuances of each AVM may be more influential than previously considered. This challenges the assumption that DVD universally raises surgical risk, highlighting a potential need for further stratification within the Spetzler-Martin grading system, particularly for intermediate-grade AVMs.

### Limitations and future directions

While the study presents notable findings, it has limitations that warrant discussion. First, the small number of outcome events (*n* = 14) limits the number of covariates that can be reliably included in multivariable models; we addressed this by employing Firth-penalized logistic regression and a parsimonious modeling strategy, but confidence intervals remain wide and more complex models were avoided due to the risk of overfitting. Second, as a retrospective study including only surgically treated patients, it may be subject to selection bias; AVMs with DVD may have been preferentially triaged to non-surgical management, potentially underestimating the true risk associated with DVD. All cases were performed at high-volume tertiary care centers with established cerebrovascular programs. However, surgical techniques and perioperative management protocols were not standardized across sites, potentially introducing inter-center variability.

Consecutive enrollment was not necessarily present at all sites. Structural confounding between DVD and overall lesion complexity cannot be fully excluded despite adjustment for SM Grade III. The sample size limits subgroup analyses, particularly within individual SM grades. The median follow-up of 13 months may not capture delayed neurological recovery, particularly in younger patients. Future studies with prospective designs and larger cohorts are needed to validate these findings.

For borderline resection cases, the association between DVD and poor outcomes should be weighed alongside other clinical factors when considering intervention. Additionally, neurosurgeons could consider DVD as part of a comprehensive surgical approach, leveraging modern microsurgical techniques to manage these lesions with lower expected morbidity. 

Incorporating new metrics for venous drainage volume or outflow resistance could enhance AVM surgical stratification and reflect modern neurosurgical capabilities. As surgical technology evolves, revisiting and updating traditional grading frameworks may enable more precise predictions of patient outcomes, thereby optimizing AVM management and improving long-term neurological results. Additionally, given the high risk associated with DVD, transvenous embolization may offer a safer alternative or adjunct to microsurgery. In cases with a compact nidus and a single deep venous outflow, TVE avoids deep vein sacrifice, may reduce morbidity, and should be explored further in treatment algorithms for DVD-dominant AVMs [24]. 

## Conclusions

DVD was independently associated with worse functional outcomes after resection of Spetzler-Martin Grade II-III AVMs in Firth-penalized logistic regression, adjusting for age and pre-existing functional disability, with approximately a seven-fold increase in odds. This supports continued attention to venous drainage patterns in preoperative risk assessment, particularly as modern surgical techniques continue to advance. Integrating these insights into risk assessment models could refine patient selection and enhance preoperative counseling, ultimately contributing to more individualized and effective management strategies for intermediate-grade AVMs.

## Data Availability

Limited data are available from the corresponding author upon reasonable request.

## Abbreviations

AVM: arteriovenous malformation
mRS: modified Rankin Scale
SM Grade: Spetzler-Martin Grade
DVD: deep venous drainage
OR: odds ratio
CI: confidence interval
EPV: events per variable
VIF: variance inflation factor
STROBE: Strengthening the Reporting of Observational Studies in Epidemiology
IRB: Institutional Review Board
TVE: transvenous embolization
